# Supplementary material for: A high-resolution mRNA expression time course of embryonic development in zebrafish
Source: eLife. 2017 Nov 16;6:e30860. doi: 10.7554/eLife.30860 (PMC5690287; doi:10.7554/eLife.30860)
Supplement: Supplementary file 6. [file elife-30860-supp6.zip › biolayout-clusters-files/Cluster250-genes.html]

Cluster250


# Cluster250: Genes

| Ensembl ID | Gene Name | Chr | Start | End | Biotype |
| --- | --- | --- | --- | --- | --- |
| ENSDARG00000103086 | BX901974.2 | 4 | 62148965 | 62150080 | protein\_coding |
| ENSDARG00000044355 | CT030712.1 | 9 | 24897579 | 24899212 | protein\_coding |
| ENSDARG00000018404 | krt18 | 23 | 10412881 | 10416784 | protein\_coding |
| ENSDARG00000100630 | lye | 19 | 17461686 | 17463682 | protein\_coding |
| ENSDARG00000037429 | tll1 | 1 | 20040165 | 20133086 | protein\_coding |
| ENSDARG00000056324 | zgc:123295 | 3 | 21059218 | 21068196 | protein\_coding |
